# Conspecific Presence Improves Episodic-Like Memory in Rats

**DOI:** 10.3389/fnbeh.2020.572150

**Published:** 2021-01-15

**Authors:** Maria Augustta Sobral de França Malheiros, Rochele Castelo-Branco, Paulo Henrique Santos de Medeiros, Pedro Emmílio de Lima Marinho, Ywlliane da Silva Rodrigues Meurer, Flávio Freitas Barbosa

**Affiliations:** ^1^Laboratory of Psychopharmaology, Federal University of Paraíba, João Pessoa, Brazil; ^2^Memory and Cognition Studies Laboratory, Department of Psychology, Federal University of Paraíba, João Pessoa, Brazil

**Keywords:** episodic-like memory, social environment, exploration, Wistar rats, anxiety-like behavior

## Abstract

A number of studies have provided evidence that animals, including rats, remember past episodes. However, few experiments have addressed episodic-like memory from a social perspective. In the present study, we evaluated Wistar rats in the WWWhen/ELM task as single setups and in dyads, applying a long retention interval. We also investigated behaviors that could subserve the emergence of this type of memory. We found that only rats tested in the social setting were able to recollect an integrated episodic-like memory that lasted 24 h. Additionally, rats in dyads presented higher levels of exploration during the task. When exposed to the testing environment, the dyads exhibited affiliative behavior toward each other and presented fewer anxiety-like responses. Our findings indicate that the presence of a conspecific could act as a facilitating factor in memory evaluations based on spontaneous exploration of objects and provide empirical support for applying more naturalistic settings in investigations of episodic-like memory in rats.

## Introduction

The capacity to encode, store and recall life events, referred to as episodic memory, is a significant cognitive ability that humans have acquired through evolution. According to Tulving, this is a type of memory that deals with the recollection of experiences that occurred at particular places at particular time points, thus relating to the *what*, *where*, *and when* of a specific event (Tulving, 1972; Tulving, 2002).

Although the traditional concept of episodic memory was restricted to human beings, several studies have provided evidence that other animals also display high order memory for single experiences. They were able to demonstrate that birds (Clayton and Dickinson, 1998; de Kort et al., 2005) and rodents (Ergorul and Eichenbaum, 2004; Dere et al., 2005; Kart-Teke et al., 2006) may meet the behavioral criteria for episodic memory, mainly featured as the ability to integrate and remember the “what,” “where,” and “when” attributes of specific past events.

In rodents, episodic-like memory (ELM) has been studied in different experimental designs, but most of them explore the novelty-preference paradigm in memory assessments for trial-unique events (Dere et al., 2005; Kart-Teke et al., 2006; Drieskens et al., 2017; de Souza et al., 2019). A prominent line of investigation is the test designed by Dere et al. and Kart-Teke et al., a protocol that combines three different tasks that separately evaluate the elements *what-where-when* in an integrative task (WWWhen/ELM task), which is hippocampal-dependent, likewise human episodic memory (Dere et al., 2005; Kart-Teke et al., 2006; Drieskens et al., 2017).

Ever since its introduction, most protocols of episodic-like memory evaluate animals individually, overlooking the possible impact of social learning on the performance in the task. From an ethological perspective, this approach might have some consequences to the research findings, considering that the experimenters are dealing with a social species. Learning with each other composes an important process of cognitive function for social animals, with clear adaptive value. The behavior of animals in groups (dyads or more) provides a rich source of information that animals may use to improve their behavior performance. It is well known that the presence of conspecific animals affects the performance of several tasks in many species, such as humans and non-humans primates, birds, and rodents. For instance, it was shown that the social context altered the spatial cognition of rats tested for free exploration in an open field, as they traveled faster, covered a greater area and took wider turns when arranged in dyads than when alone (Weiss et al., 2015).

A social facilitation effect was also described for mice evaluated in a memory assessment, given that animals tested in the company of a cagemate had a superior performance in the spatial object recognition task than individually tested subjects. Furthermore, the authors observed that animals tested in pairs presented more rearing, which is considered a behavioral parameter of exploration in rodents. Additionally, joint habituation to the open field reduced the repetitive self-grooming of autistic-like BTBR mice investigated in the same work (Lipina and Roder, 2013). In this context, experiments with rats’ working memory also showed that the spatial choices made by a rat affected the choices made by its familiar conspecific (Brown et al., 2007, 2008). Additionally, a study of Hughes addressed the role of the social environment as an emotional modulator in rats. The author compared the locomotion and exploration in rats inserted alone or in pairs in an illuminated open field, and verified that the presence of a conspecific reduced defecation and freezing (Hughes, 1969).

The findings of the literature reviewed above demonstrate that rodents respond to the social context, considering that they behave differently to how they would have behaved individually under identical conditions. It is important to note that, in rats, there is also evidence of more complex processes than social facilitation, like observational learning, cooperation and empathy (Rutte and Taborsky, 2007; Viana et al., 2010; Bartal et al., 2011; Wood et al., 2016; Weiss et al., 2017). Therefore, the social nature is an important dimension of the rat’s behavior, even though this is frequently an overlooked factor in the field of episodic-like memory. Since the output of the ELM assessment is inferred from the animal’s behavior, we assumed that the presence of a conspecific would promote a positive environment for rats in the experimental setting and enhance the subjects’ performance in the task. We also hypothesized that joint habituation would decrease the aversiveness of the open field, and this reduced sense of risk would be carried over to the experiment, supporting the animals in the processes required for the construction of episodic-like memory. Moreover, to our knowledge, excluding the aforementioned study of Lipina and Roder with mice (Lipina and Roder, 2013), investigations that directly measures the influence of the social context in the actual experimental setting of object recognition memory assessments are absent in the literature. There is evidence that animals raised in isolation present deficits in object recognition test (ORT) (Bianchi et al., 2006), or that social housing, compared to single housing, leads to weaker memory consolidation in a novel recognition task (NOR), probably because of the interference from socialization in the intertrial interval (van Goethem et al., 2012). However, in those studies, rodents were tested individually, which limits the scope of interpretation concerning the social effect. In addition to that, we should consider that object recognition memory tasks and the WWWhen/ELM task, although being similar in structure, assess different memory systems and present different cognitive demands.

In the present study, we evaluated rats in the WWWhen/ELM task as singles and dyads, applying a long retention interval. To date, all demonstrations of episodic-like memory in rodents using this protocol applied 50–60 min inter-trial interval between training and test (Dere et al., 2005; Kart-Teke et al., 2006; Drieskens et al., 2017; de Souza et al., 2019). Barbosa et al. (2012) demonstrated that rats recall some components of ELM using a similar experimental design in a 24 h interval, but Chao et al. (2014) showed that rats are unable to recollect an ELM in a delay 23 h applying the WWWhen/ELM. Previous studies from our lab confirmed this information (unpublished results). Thus, we hypothesized for the first time an effect of the social context on episodic-like memory. In this regard, we predicted that the presence of another conspecific would facilitate the persistence of the episodic-like memory in a 24 h retention interval, and we followed up the question of whether the social context would improve the performance of rats in this task by attenuating anxiety and stimulating exploration.

## Materials and Methods

### Animals

Twenty-six male Wistar rats (3–4 months old, weighing between 240 and 365 g)– supplied by the vivarium Prof. Thomas George from Biotechnology Center of the Federal University of Paraíba (IPeFarM-UFPB) –were housed four or five per cage in a room with controlled temperature (24 ± 1°C) and a 12:12 light/dark cycle (lights on at 6 a.m.). Water and food were available *ad libitum*. All procedures were approved by the local ethics committee (protocol N° 092/2015) and followed the guidelines of the Brazilian law for the use of animals in research (Law N° 11.794/2008).

### Apparatus and Objects

The animals performed the task in a sound-attenuated room, with a masking noise and controlled light intensity. For the WWWhen/ELM task, we used a circular open field measuring 60 cm in diameter and 45 cm in height, with a black surface covering the inside floor, and surrounded by distant and proximal cues in the walls. We used four sets of objects in quadruplicate, for which we have ruled out any bias toward specific objects, according to previous studies from our lab (Drieskens et al., 2017; de Souza et al., 2019). The objects were made of plastic and differed in terms of height, color, shape (height: 5–15 cm; width: 5–10 cm) and had sufficient weight to ensure that the animals would not be able to displace them. All objects and spatial locations in the open field were randomized among animals and groups. At the end of each trial, the apparatus and objects were thoroughly cleaned with 5% alcohol. The sessions were recorded by a digital camera (HD Webcam C270, Logitech Inc., CA, United States) positioned 160 cm above the apparatus, and plugged in real time by an video-recording software (Debut Video Capture, NCH Software, Inc., United States) installed in a computer in a separate room, from where the experimenter monitored the sessions. The behavioral parameters were scored off-line from video recordings and analyzed by the tracking software Ethowatcher (Crispim Junior et al., 2012) and ANY-maze (Stoelting Co, Wood Dale, IL, United States). For total distance traveled, we used the Id Tracker toolbox (Pérez-Escudero et al., 2014).

### Experimental Procedures

**FIGURE 1:**
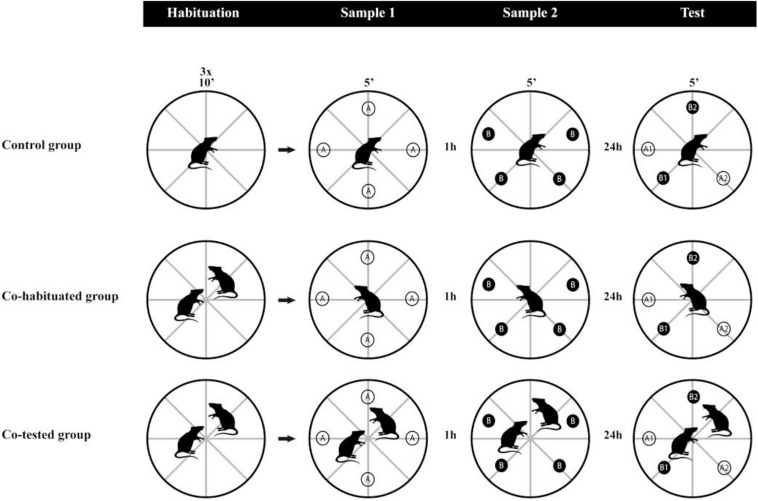
Experimental design. Three groups of rats performed habituation to the apparatus, followed by the WWWhen/ELM task. The control group went through habituation sessions and the WWWhen/ELM task always in single setups. The Co-Hab group performed the habituation sessions in dyads but conducted the WWWhen/ELM task in single setups. The Co-Test group performed both habituation and the WWWhen/ELM task in dyads. The WWWhen/ELM task consists of two sample trials and a test session of object exploration. Compared to samples, objects could be stationary (A1 and B1) or in different locations (A2 and B2). Animals tend to explore A1 > B1 (“temporal pattern”), B2 > B1 (“spatial pattern”) and A1 > A2 (“integrative pattern”). A1: old stationary; A2: old displaced; B1: recent stationary; B2: recent displaced.

All procedures were conducted during the light phase (from 10:00 a.m. to 4:00 p.m). To assess whether the social context affects the rats’ performance in the WWWhen/ELM task, we randomly allocated the rats to one of three groups: (1) Control group (**Control;** *n* = 9) – animals were placed individually in the open field for the habituation sessions and the task trials; (2) Co-habituated dyad group (**Co-Hab**; *n* = 8) – performed the habituation sessions in dyads, but the animals were tested individually in the sample and test sessions; (3) Co-tested dyad group (**Co-Test**; *n* = 8) – went through all the procedures in dyads (Figure 1). Firstly, all rats were handled for 15 min/day for 5 days before the start of the habituation. Then, all rats were submitted to the tube test (described in Section Assessment of Social Dominance) for social dominance evaluation and individual identification. After that, the animals underwent three daily sessions of habituation to the apparatus, which lasted 10 min each and consisted of placing the rats in the empty open field and let them explore the surroundings. Twenty-four hours later, we started the protocol of the WWWhen/ELM task, adapted from the task previously designed by Kart-Teke et al. (2006), according to Drieskens et al. (2017) and de Souza et al. (2019). The task consisted of two sample trials and only a test trial of 5 min each. In the first sample trial, four identical objects (**A**) were arranged in the open field. The second sample trial was carried out 1 h later, in which four identical objects, different from those already presented to the animals (**B**), were provided. Two of these objects remained at spatial coordinates already occupied by A objects. Lastly, after 24 h, the test trial comprised four objects, all of them already presented in the previous sessions, in which two objects maintained stationary positions in the apparatus (A1 and B1) and two were displaced (A2 and B2). Throughout the experimental procedures, rats were removed from their home cages individually or in dyads only to perform the WWWhen/ELM task in the open field, returning afterward to their initial housing conditions.

According to this model, rats explore recent displaced object (B2) more than recent stationary object (B1), indicating the spatial aspect of memory. For the temporal aspect of memory, rats tend to explore old stationary objects more than recent stationary objects (A1 > B1). The episodic-like memory integration can be inferred when the subjects explore “A1” more than “A2,” which implies that rats are able to concomitantly discriminate whether the objects were spatially displaced or stationary compared to their first appearance in the task. As we mentioned before, in order to investigate not only the effect of the social environment but also the strength of this variable on the rats’ performance, we decided to increase the delay between sample two and test phase to 24 h, which until now had not yet been evaluated (Figure 1).

#### Assessment of Social Dominance

Prior to the first habituation session, we investigated social dominance in rats applying the tube test, according to Cao et al. (2017). In this evaluation, each rat of the dyad was released in the opposite ends of a transparent tube whose diameter fits only one individual. As stated by Cao et al. (2017), the dominant rat is the one that forces the other rat out of the tube. All pairs consisted of one subordinate and one dominant animal, and we kept the same individuals in each dyad during the task phases (i.e., we did not alter the dyad-mate of the rats, so each individual was always kept in a certain dyad, and was re-tested in Sample 1, Sample 2 and Test). After this procedure, we colored dominant and subordinate rats with blue and red ink, respectively, on the back, for tracking in video analysis. Given that each dyad consisted of one dominant and one subordinate animal, each social dominance category (dominant or subordinate) contain half of the rats of each dyad group.

#### Object Exploration

Object exploration was registered when the animal was touching the objects with its snout and/or forepaws or when the rat’s snout was within 2 cm of the object, while turning around or sitting on the object was not considered exploration, in agreement with several authors (DeVito and Eichenbaum, 2010; van Goethem et al., 2012; Inostroza et al., 2013). We calculated discrimination indexes (DI) for each expected pattern of exploration. This variable indicates how much an object is discriminated relative to another object. Thus, for the *temporal index* we used the formula: (time exploring A1 – time exploring B1)/(time exploring A1 + time exploring B1); for the *spatial index* we used the formula: (time exploring B2 – time exploring B1)/(time exploring B2 + time exploring B1) and for the *integration index* we used the formula: (time exploring A1 – time exploring A2)/(time exploring A1 + time exploring A2). Positive values of the spatial, temporal, and integrative indices represent the expression of memory for object location, temporal order, and ELM integration. The analysis of object exploration was conducted by an experimenter blind to the WWWhen/ELM task trials and blind to object condition. We also measured the time spent in each object, the total time and frequency of object exploration, as well as the frequency and latency to visit the first object explored by the animals in the experiment during the test session.

#### Anxiety-Like and Social Behavior Measures

In order to assess anxiety-like responses, we measured the time that each rat spent self-grooming in the open-field and thigmotactic responses in the first habituation session, which is expected to be the most stressful condition due to the environmental novelty (Choleris et al., 2001). Moreover, we also recorded the interactions exhibited by the dyads during this session, to evaluate whether the emergence of social behaviors is in line with the attenuation of anxiety-like responses. Therefore, we recorded the frequency of affiliative (e.g.,: huddling, sniffing, allogrooming, defined according to Barnett, 1976, 2009a) and agonistic behaviors (e.g.,: components of conflict between males, defined according to Barnett, 1976, 2009b), as well as the time in which there was no interactions between the animals in the dyads. In addition to the analyses carried out in the first habituation session, we registered social behaviors in the test session of the WWWhen/ELM task, in order to verify the interaction between conspecifics during memory evaluation.

### Statistical Analysis

The normality of distribution and homogeneity of variances was confirmed using the Shapiro-Wilk and Levene’s test. After that, we adopted parametric or non-parametric tests according to specific analysis of our data regarding assumptions criteria. We conducted repeated measures one-way analysis of variance (ANOVA) to compare the total exploration time, frequency to visit objects and the distance traveled in the open field along the experimental session A multivariate ANOVA (MANOVA) with discrimination indexes (Temporal, Spatial, and Integration) as the within-subject factor and groups (Control, Co-Hab, and Co-Test) as the between-subject factor was performed to evaluate the combined effect of discrimination indexes among the groups. A Friedman test was conducted to compare the frequency on the first object visited along the experimental sessions. Wilcoxon’s signed rank test with Bonferroni correction *a posteriori* were used to compare the first object exploration among the experimental sessions. A MANOVA with exploration time for each object (A1, A2, B1, and B2) as the within-subject factor and groups (Control, Co-Hab, and Co-Test) as the between-subject factor was performed to evaluate the combined effect of exploration time of each individual object among the groups. Additionally, Wilcoxon’s signed rank test with Bonferroni correction were used to check for differences in the exploratory pattern A1 > A2, B1 > B2 and A1 > B1 of the WWWhen/ELM task among the groups. One-sample t-statistics were performed to assess whether the discrimination indexes were different from zero, since random exploration would result in equal exploration of both objects. Additionally, one-way ANOVA were performed to evaluate the frequency and latency to visit objects between groups during the test trial, as well as the time spent in self-grooming during the first habituation session. In order to reduce the risk of errors inflation from performing separate analyses of variance, a one-way univariate and multivariate analysis of covariance (ANCOVA and MANCOVA, respectively) was used to examine differences in exploration time and discrimination indexes, respectively, among the groups (Control, Co-Hab, and Co-Test) whilst controlling hierarchical status (dominant or subordinate) during the test trial. The effect size for *t*-tests and for the ANOVAs were calculated using G^∗^Power 3 (Faul et al., 2009). We also applied Wilcoxon signed rank test to examine the social behaviors displayed by the rat dyads [Co-Hab (*n* = 8); Co-Test (*n* = 6)], as data did not show a normal distribution. We evaluated the individual performance of each rat of a dyad group. However, all comparisons between groups were made using average of performance in the WWWhen/ELM task. The performance of the experimental groups in the WWWhen/ELM task were expressed, when appropriated, as mean ± Standard error of the mean (SEM) or median ± interquartile range (IQR). Effects were considered significant when *p* ≤ 0.05 and we used two-tailed tests.

## Results

### Discrimination Indexes

**FIGURE 2:**
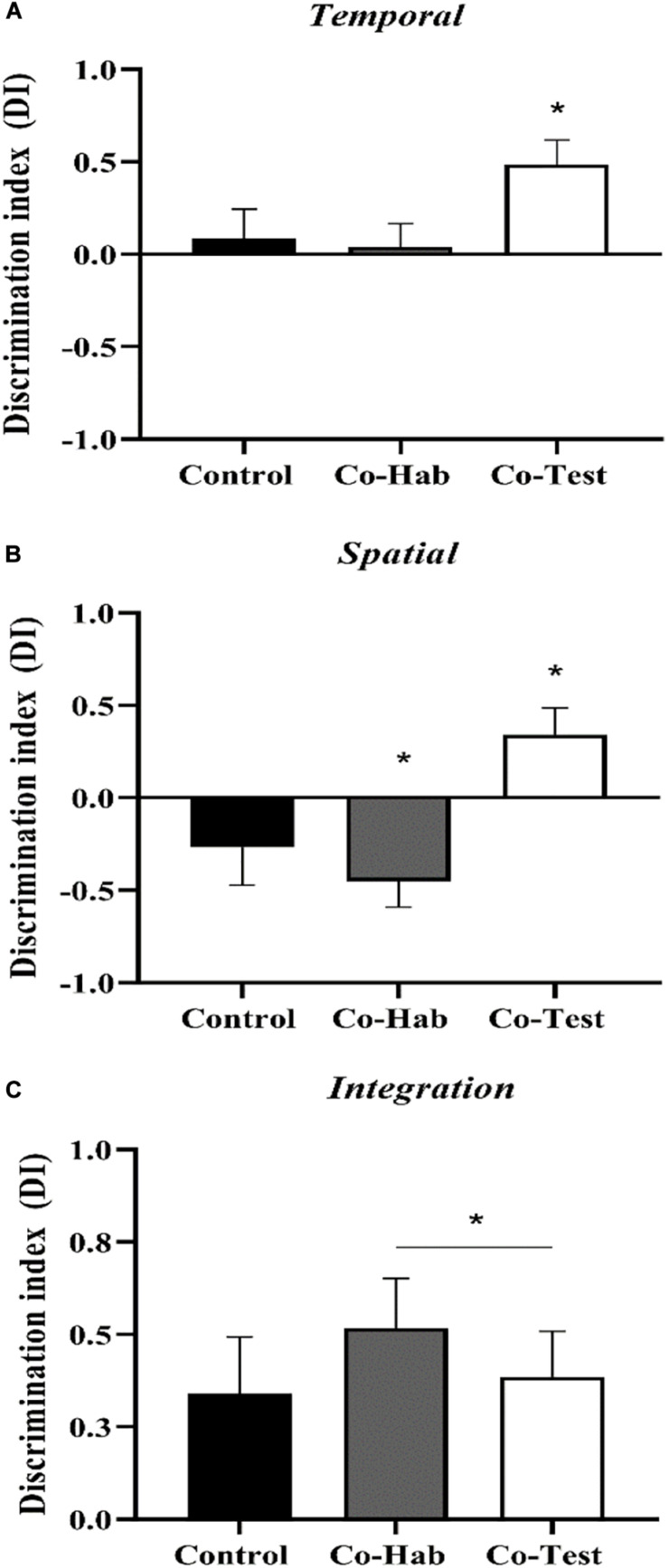
The effects of the social context on the WWWhen/ELM task. Discrimination indexes for **(A)** Temporal, **(B)** Spatial, and **(C)** Integration components of ELM for Control, Co-Hab and Co-Test experimental groups. Only the rats in the Co-Test group presented episodic-like memory (i.e.,: only the Co-Test group recollected an integrative memory for “what,” “where,” and “when,” since it showed positive discrimination indexes for the spatial, temporal, and integration elements of ELM) (*p < 0.05 comparison against zero chance level using one-sample *t*-test). Data represent mean ± SEM.

To evaluate the effect of the social context on discrimination indexes of the WWWhen/ELM task, Shapiro-Wilk and Levene’s tests were carried out to check the normality and homogeneity of variances, and the assumptions met. Then, a multivariate ANOVA was conducted and revealed a marginally significant main effect for the groups [*F*_(__6_,_42__)_ = 2.182, *p* = 0.064; η^2^ = 0.238; Pillai’s trace: 0.475]. A *posteriori* analysis showed no differences between the Control group versus Co-Hab and Co-Test group for *Temporal* DI (*p* = 0.99; *p* = 0.15; respectively). For *Spatial* DI, Control group showed a lower discrimination index than the Co-Test group (*p* = 0.05) but not compared to Co-Hab group (*p* = 0.833) in the test trial. There were no differences among the groups on indexes values of *Integration* DI (*p* > 0.05). Nevertheless, the results of the discrimination indexes were also compared against chance level through one-sampled *t*-tests, since we assume episodic-like memory when the animals exhibit all components of WWWhen/ELM task. The control group presented marginally significant differences for the integration aspect [*t*_(__8__)_ = 2.197, *p* = 0.059; *d*’ = 0.73], but not for spatial [*t*_(__8__)_ = –1.295, *p* = 0.232; *d*’ = –0.43] and temporal [*t*_(__8__)_ = 0.548, *p* = 0.599; *d*’ = 0.18] aspects. The Co-Hab group presented significant differences for two aspects: spatial [*t*_(__7__)_ = –3.244, *p* = 0.014; *d*’ = –1.14] and integration [*t*_(__7__)_ = 3.840, *p* = 0.006 d’ = 1.35]; not for the temporal aspect [*t*_(__7__)_ = 0.319, *p* = 0.759; *d*’ = 0.11]. For the spatial index, both control and Co-Hab groups showed an inverted pattern. The Co-Test group presented statistically significant differences for three aspects, as follows: temporal [*t*_(__7__)_ = 3.728, *p* = 0.007; *d*’ = 1.32], spatial [*t*_(__7__)_ = 2.372, *p* = 0.049; *d* = 0.84] and integration [*t*_(__7__)_ = 3.121, *p* = 0.017; (*d*’ = 1.10]; see the Supplementary Material). Thus, only the Co-Test group recollected an integrated episodic-like memory in the WWWhen/ELM task (Figure 2). Together, these data indicate that Control and Co-Hab groups were significantly impaired in episodic-like memory ability, as tested in the what-where-when task.

### Object Exploration

Given that rats exhibit biased exploration, in terms of spending more time exploring the old familiar object (A1) than the old displaced object (A2) in the WWWhen/ELM protocol with a retention interval of 50 min (for a review, see Chao et al., 2020), we evaluated the exploration time of each object among the groups in the test trial, after a retention interval of 24 h. Multivariate ANOVA with exploration time for each object (A1, A2, B1, and B2) as the within-subject factor and groups as the between-subject factor revealed that there were no significant differences in exploration time of each object among the groups [*F*_(__8_,_40__)_: 1.088; *p* = 0.391; η^2^ = 0.179; Pillai’s trace = 0.358], which suggests that we can rule out effects such as a lack of drive to explore the objects or object preference throughout the test trial. In agreement, to evaluate the total exploration time of objects along experimental sessions, a repeated measure one-way ANOVA revealed session [*F*_(__2_,_44__)_: 19.576; *p* < 0.001; η^2^ = 0.471], group [*F*_(2, 22)_: 3.748, *p* = 0.040, η^2^ = 0.254], but not “group versus session” interaction effects [*F*_(__4_,_44__)_: 1.368; *p* = 0.260, η^2^ = 0.111]. Tukey’s *post hoc* comparison for group main effect indicated that the Co-Test group had a longer total time of object exploration in all sessions when compared to the control group [*p* = 0.040; Supplementary Material] (Table 1).

**TABLE 1:** The effects of the social context on the WWWhen/ELM task.

	**Control**	**Co-Hab**	**Co-Test**
**Total exploration time (s)**			
Sample 1	33.30 ± 4.46	36.78 ± 2.86	45.57 ± 5.05
Sample 2	22.92 ± 4.41	35.59 ± 3.51	38.78 ± 4.64
Test	17.51 ± 2.67^#^	25.38 ± 4.81^#^	21.89 ± 2.90^#^
**Total distance (m)**			
Sample 1	1.42 ± 0.91	1.73 ± 0.88	1.88 ± 0.72
Sample 2	1.22 ± 0.93	1.36 ± 1.08	1.37 ± 1.14
Test	1.10 ± 1.47	1.24 ± 1.52	1.08 ± 1.10
**Frequency to visit the objects**			
Sample 1	26.00 ± 2.39	34.50 ± 3.43	33.00 ± 3.58
Sample 2	18.22 ± 3.28	25.75 ± 1.39	26.87 ± 3.32
Test	14.55 ± 2.11^#^	21.37 ± 2.39^#^	15.87 ± 1.88*
**Frequency to visit the first object explored by the rats**			
Test	4.88 ± 0.42^#^	5.75 ± 1.29	3.25 ± 0.45*
**Latency to visit the first object explored by the rats**			
Test	3.81 ± 0.76	2.31 ± 0.42	4.39 ± 0.83

Total object exploration time (seconds), total distance traveled in the open field (meters), frequency of visits to the objects (number of visits), latency to visit the first object in the test trial (time in seconds), frequency of visits to the first object explored in the test trial (number of visits) for Control, Co-Hab, and Co-Test groups along the experimental session. The Co-Test group showed a higher level of exploration in the experimental session (*p < 0.05 compared to Sample 1 and Sample 2; #*p* < 0.05 compared to Sample 1). All groups showed a time reduction in exploration along the experimental session. Data are represented as mean ± SEM.

**FIGURE 3:**
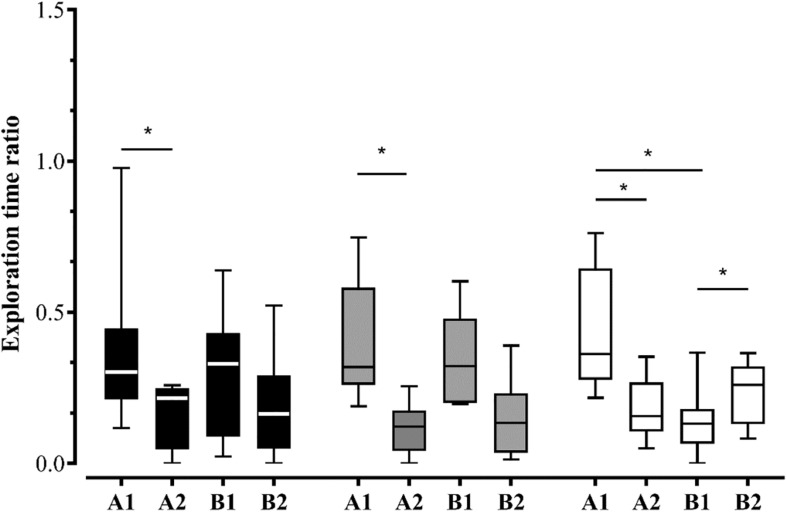
The effects of the social context on the WWWhen/ELM task. Exploration time ratio of each object – A1, A2, B1, and B2 – for Control, Co-Hab and Co-Test groups during test session. The Co-Test group exhibited the exploratiordsn pattern that defines WWWhen/ELM, i.e.,: they explored the stationary object more than the displaced one if they were presented earlier, while simultaneously exploring the displaced object more than the stationary one if they were presented recently. However, the Control and Co-Hab groups did not show this pattern. *p < 0.05 for comparison between biased exploratory pattern A1 > A2, A1 > B1, and B2 > B1 of the WWWhen/ELM task (Wilcoxon signed-rank test, Bonferroni corrected). The graphs represent the median ± interquartile range (IQR).

Also, we evaluated the exploration patterns of A1 > B1 (*when*: old familiar over old recent stationary objects), B2 > B1 (*where:* novel location over old one in recent familiar objects) and A1 > A2 (old location over novel one in old familiar objects, that indicates an interaction between object-location and temporal-order in the WWWhen/ELM task and, hence episodic-like memory in animals) (for review Chao et al., 2020). Given that the exploration time of objects by groups alone did not meet normality, we conducted Wilcoxon signed-rank test with Bonferroni corrections to compare the exploration patterns. Our results showed that Control group did not exhibit biased exploration pattern A1 > B1 [*z* = –0.652; *p* = 0.515], B2 > B1 [*z* = –1.007; *p* = 0.314], and A1 > A2 [*z* = –1.955; *p* = 0.051]. Nevertheless, the Co-Hab group exhibited biased exploratory pattern A1 > A2 [*z* = –2.521; *p* = 0.012], but not B2 > B1 [*z* = –1.820; *p* = 0.069] and A1 > B1 [*z* = –0.280; *p* = 0.779]. On the other hand, Co-Test group showed all biased exploratory pattern: A1 > B1 [*z* = –2.240; *p* = 0.025], B2 > B1 [*z* = –1.960; *p* = 0.050] and, A1 > A2 [*z* = –2.100; *p* = 0.036], which provides evidence that the Co-Test group showed an integrated memory for “what,” “where,” and “when,” i.e.,: ELM (Figure 3).

### Total Distance Traveled

Concerning the animals’ locomotion in the open field, the repeated measures one-way ANOVA revealed no main effect for the habituation sessions [*F*_(__2_,_44__)_ = 3.071; *p* = 0.070; η^2^ = 0.122, considering the Greenhouse-Geisser estimate], group [*F*_(__2_,_22__)_ = 1.962; *p* = 0.164; η^2^ = 0.151], and “group versus habituation sessions” interaction [*F*_(__4_,_44__)_ = 0.786; *p* = 0.515; η^2^ = 0.067]. For the experimental session, there was a main effect for session [*F*_(__2_,_44__)_ = 27.236; *p* < 0.001; η^2^ = 0.553], but not group [*F*_(__2_,_22__)_ = 1.925; *p* = 0.170; η^2^ = 0.149], or “sessions versus group” interaction effects [*F*_(__4_,_44__)_ = 2.048; *p* = 0.104; η^2^ = 0.157] (Supplementary Material). Tukey’s *post hoc* comparisons showed that rats had a higher distance traveled in the sample 1 compared to the other trials, which suggest habituation to the arena along the experimental session.

### Frequency and Latency to Visit the Objects

To evaluate possible interference (either preference or avoidance toward each object), we first evaluated the frequency to visit all objects exhibited by the animals along the experimental session (Supplementary Material). A repeated measure one-way ANOVA revealed group [*F*_(__2_,_22__):_5.322; *p* = 0.013; η^2^ = 0.326], session [*F*_(2,44)_: 20.166; *p* < 0.0001; η^2^ = 0.478], but not “group versus session” interaction [*F*_(__4_,_44__)_: 0.611; *p* = 0.657; η^2^ = 0.053] effects. Sidak’s correction for multiple comparisons showed that rats had a significant difference on the frequency of visits to the objects along the experimental session. Both Control and Co-Hab groups showed a lower frequency to visit the objects in the test trial compared to sample 1 [*p* < 0.05], but not compared to sample 2 [*p* > 0.05]. On the other hand, the Co-Test group showed a lower frequency to visit the objects in the test trial when compared to both sample 1 and sample 2 [*p* < 0.001; *p* = 0.005; respectively] (Table 1).

We also evaluate the frequency of visits to the first object explored by the animals along the experimental session. Because Levene’s test and normality checks were carried out and the assumptions did not met, a non-parametric alternative to the one-way ANOVA with repeated measures was conducted. A Friedman one-way test indicated that frequency of visits to the first object were rated differently by groups along experimental sessions, *X*^2^(2) = 15.732, *p* < 0.001. Median for frequency of visits to the first object explored by rats were for Control group (Sample 1 = 9.00, Sample 2 = 6, Test = 5), Co-Hab group (Sample 1 = 9.00, Sample 2 = 7.50, Test = 5.50) and Co-Test group (Sample 1 = 9.50, Sample 2 = 7.50, Test = 3.00). *Post hoc* analysis with Wilcoxon signed-rank tests was conducted with a Bonferroni correction applied, revealing that rats had a significant difference on the frequency of visits to the first object explored along the experimental sessions. The Control group showed a lower frequency to visit the first object in the test trial compared to sample 1 [*p* = 0.004], but not compared to sample 2 [*p* > 0.05]. The Co-Hab group did not exhibit difference [*p* = 0.657] on the number of visits to the first object along the experimental session. On the other hand, the Co-Test group showed a lower frequency of visits to the first object in the test trial when compared to both sample 1 and sample 2 [*p* = 0.026; *p* = 0.026; respectively] (Table 1). These data imply that the animals did not develop a preference for objects due to the first exploration episode. In addition, we evaluated, in the test trial, the latency to visit the first object explored by the animals in the experimental session. Accordingly, a one-way ANOVA revealed no differences on the latency to visit the first object explored by the animals between groups in the test trial [*F*_(__2_,_22__)_: 2.232; *p* = 0.131, η^2^ = 0.169] (Table 1).

### Social Dominance

A major concern regarding interpretation of behavioral data in a social context is whether social hierarchy might eventually interfere in ELM. To evaluate a possible effect of hierarchical status on total exploration time along the sessions, we conducted a repeated measure ANCOVA to compare the time exploring objects along sessions between groups whilst controlling the social hierarchical status. Levene’s test and normality checks were carried out and the assumptions met. Mauchly’s test of sphericity indicated that the assumption of sphericity had not been violated (*X*^2^ = 2.245, *p* = 0.325). Regarding exploration time, we found a statistically significant result for sessions [*F*_(__2_,_42__)_: 7.475; *p* = 0.002; η^2^ = 0.263], but not for “sessions versus group” [*F*_(__4_,_42__)_ = 1.236, *p* = 0.310, η^2^ = 0.105], neither for the “sessions versus hierarchy” interaction [*F*_(__2_,_42__)_: 0.914; *p* = 0.409; η^2^ = 0.042]. Thus, we found that dominant and subordinate animals behaved similarly in relation to the exploration of the objects.

We then compared the DI between groups whilst controlling the social hierarchy to evaluate the effects on components of episodic-like memory. An initial MANCOVA using Pillai’s test examined the influence of social hierarchy, and revealed a marginal effect for group [*F*_(__6_,_40__)_: 2.041; *p* = 0.082; η^2^ = 0.152; Pillai’s trace: 0.469], but not for social hierarchy [*F*_(__3_,_19__)_: 1.135; *p* = 0.360; η^2^ = 0.152; Pillai’s trace: 0.152]. Thereafter, an univariate ANOVA for discrimination indexes between groups whilst controlling the social hierarchy predicted effects Spatial DI [*F*_(__2_,_21__)_: 5.480; *p* = 0.012; η^2^ = 0.343], marginally significant for Temporal DI [*F*_(__2_,_21__)_: 2.891; *p* = 0.078; η^2^ = 0.216], but not on Integration DI [*F*_(__2_,_21__)_: 0.323; *p* = 0.728; η^2^ = 0.030], which suggest that the effects of social context on components of ELM occurs in a social hierarchy-independent manner (Supplementary Material).

### Self-Grooming Behavior

Because habituation to the testing arena is used to reduce anxiety and arousal levels in animals, we investigated the self-grooming behavior between groups as a measure to infer about anxiety levels in the animals during the first habituation session. A one-way ANOVA revealed a significantly main effect for group [*F*_(__2_,_22__)_ = 4.034; *p* = 0.032] on the time rats spent performing self-grooming during the first session of habituation in the open field. Although Tukey’s *post hoc* comparison did not revealed differences among the groups, we then conducted power analysis to evaluate the effect size between groups. We found a large effect sizes for comparisons between the control group and both the Co-Hab group (*d*’ = 1.31) and the Co-Test group (*d*’ = 1.07). There was a small effect size for comparison between the Co-Hab group and Co-Test group (*d*’ = -0.04). Collectively, these results suggest increased self-grooming behavior in the control group, which suggest more anxiety levels in these animals (Table 2). We also checked the time spent in the center and external zone of open field along the first habituation session, but no differences were observed between groups (*p* > 0.05; Supplementary Material).

**TABLE 2:** Time spent on self-grooming and social behavior for Control, Co-Hab, and Co-Test groups during the first habituation session.

	**Control**	**Co-Hab**	**Co-Test**
**Self-grooming (s)**			
First habituation	181.53 ± 24.73	93.34 ± 20.76	96.44 ± 29.81
**Social behavior (s)**			
Affiliative	N/A	121.04 ± 27.31	189.70 ± 57.91
Agonistic	–	–	–

*There were no differences between groups. Data expressed as mean ± SEM. N/A – not applicable.*

### Social Behaviors

As hypothesized, rats in dyads expressed social behaviors toward each other in the first habituation session. There was no record of agonistic behaviors during social interactions for the rats. The animals performed huddling, allogrooming, and sniffing. In this regard, we observed that the rats from the Co-Hab group and Co-Test group spent similar time engaged in affiliative behaviors, and that the amount of time that animals spent performing social behavior was higher than the time standing alone in the open field (*z* = –3.296; *p* = 0.001; *r* = 0.88) (Table 2). As described for the first habituation session, the Co-Test group also exhibited affiliative behavior in the test session. We observed that animals explored the objects and the open field in physical contact with each other (44.48 ± 6.94) and performed sniffing (6.72 ± 2.18) and huddling (70.87 ± 30.55), but they did not engage in agonistic behavior. Thus, we found that animals in dyads performed high rates of socio-positive behaviors (*p* < 0.05; Supplementary Material).

## Discussion

In the present study, we intended to shed light on the potential factors underlying the social effect over episodic-like memories. For this purpose, we aimed to verify whether the social context influence the process of learning in rats arranged in dyads, and if these animals would outperform rats tested alone in the WWWhen/ELM task. We found that only rats belonging to the Co-Test group – those that went through all the experimental procedures with familiar conspecifics or cagemates – were able to form and recollect an integrated episodic-like memory that lasted 24 h.

Besides the results for the rats that remained in dyads, it is relevant to analyse other groups’ performance in the task. Initially, as expected, the data concerning object exploration (i.e., exploration patterns in the WWWhen/ELM) are in line with the results observed for the discrimination indexes for the three experimental groups. The Co-Hab group – in which the animals were arranged in pairs exclusively during the habituation to the apparatus – presented negative spatial discrimination index in the task, and the control group exhibited behavior in the same direction (although this result was not statistically significant). Moreover, we observed positive integration indexes in both groups. We believe that a possible explanation for these results could be interference from neophobia during the memory evaluation. Even though our data regarding object exploration (latency and frequency to approach objects) does not suggest neophobia toward novelty, it is important to consider that emotionally challenging circumstances induce different forms of anxiogenic or neophobic responses. In this context, the WWWhen/ELM task, on the whole, is based on behavioral measures of object recognition, and it is known from the NOR literature that even small amount of stress may produce a bias toward familiar objects (Ennaceur et al., 2009; Ennaceur, 2010). In this sense, we hypothesize that the rats that performed the task in isolation might have developed an adherence to object-place associations, considering their initial configuration in the task. Accordingly, for the spatial evaluation, in which the index is based on sample 02, we observed an increased exploration of the stationary object over the displaced one in the test (i.e.,: B1 > B2). As for the integration element, the index is composed of the positions occupied by the objects in sample 01 relative to the test trial, and it is possible that, in the test, the rats also avoided to dissociate the object from its original position, therefore expressing a preference for A1 over A2 (A1 > A2). It is important to note that, following this assumption, we cannot affirm that these groups actually succeeded in the Integration DI, which is a core measure for episodic-like memory and indicates that animals form an integrated representation of the two sample phases. In line with this assumption, it has been previously stated that memory tasks with a low number of trials, compared to multiple-trial tasks, are prone to behavioral variance due to stress related to handling (Ameen-Ali et al., 2015). In addition to that, in our study, not only the animals were taken in and out of the open field during the initial experimental conditions, but also they were handled again after a delay of 24 h. We should emphasize that, in these kinds of tasks, the behavioral evidence of the animals’ memory is object exploration, which is completely spontaneous. Therefore, this data requires caution in the interpretation, in order to dissociate an expression of preference toward objects – such as a neophobic response – from an actual indication of memory based on associations of place and temporal order of items explored by the animals. In this regard, we observed a salient difference in the profile of the Co-Test group, where the rats fulfilled the three aspects required for an episodic recollection. These results may indicate a potential promnestic function for the social environment.

In support of this assertion, we verified that some behavioral parameters could account for an improved ELM task performance in the social condition. Concerning anxiety-related responses, we found that, when exposed to the testing chamber for the first time, the dyad groups presented less self-grooming behavior than the rats placed alone in the open field. On the other hand, all rats presented thigmotactic responses, regardless of the experimental group. Although both measures are frequently applied for the same purpose, Ramos (2008) states that anxiety is a multidimensional phenomenon and each test assess different aspects of anxiety. Indeed, some experiments report discrepant results between grooming and other anxiety measures (e.g., Meerlo et al., 1999; Lamprea et al., 2008), and Ennaceur (2014) points out that ethological parameters not always correlate with other indexes of anxiety. In this context, Kalueff et al. (2016) claim that stressful and anxiogenic situations seem to modulate self-grooming behavior and Fernández-Teruel and Estanislau (2016) affirm that this is a useful measure of rodent anxiety-like behavior elicited by a novel environment. Therefore, based on the observed differences in this behavior, we assume that rats in dyadic couplings developed a sense of security, as proposed by Weiss et al. (2015). It is noteworthy because the original work of Kart-Teke et al. had already pointed out that the WWWhen/ELM task is susceptible to stressful stimuli (Kart-Teke et al., 2006).

Furthermore, although all the rats in our study displayed similar levels of locomotor activity, the Co-Test group spent more time exploring the objects in the task. Considering that these animals performed the ELM task in the social condition, they were the only rats that could benefit from a reduction of aversiveness due to the presence of a conspecific in the experimental phase. It is relevant to note that, besides the previous habituation to the open field, the testing environment changes again with the introduction of the objects. Given the performance of the Co-Test rats in the task, it is possible to hypothesize that those rats created a stronger memory representation of the objects due to the amount of exploration. In this sense, one might suppose that longer sample phases (for example, up to 10 min, instead of 5 min, as applied in our study) would provide an opportunity for the animals to learn about the objects more efficiently, producing similar results. However, based on what is known from object novelty memory tests, this alternative seems unlikely. Even though object exposure time is related to memory storage, according to Paes et al., the duration of the acquisition trial in these kinds of tasks is usually set to 3 or 5 min, and for rats, 5 min duration in this phase proved sufficient for reliable memory evaluation. The authors also stated that, in the case of object-in-context tasks, which share features with the WWWhen/ELM task, it is complicated to use longer acquisition trial durations, because animals may switch to a random exploration after having achieved object discrimination and recognition (Paes et al., 2018). In fact, it is reasonable to imply that the social context changes the nature of object exploration, similar to the effects of grouping on activity and exploration of rats, as a consequence of a reduced sense of risk induced by the social environment, as suggested by Weiss et al. (2015). That being the case, a lower anxiety/fearfulness profile might covary with other behaviors, such as neophobia. Neophobia constrains the approach and manipulation of objects, and the presence of group members has often been shown to facilitate exploration (Ryer and Olla, 1991). The same can be assumed for the way that animals interact with the environment during habituation to the open field – we should not expect that expanding the length of the habituation phase would be simply interchangeable by a shorter duration in the presence of a conspecific. Instead, there should be motivational factors underlying exploratory behavior that arose from the social setting.

Thus, regarding the attenuation of anxiety-like responses and an enhancement in exploratory behavior associated with the presence of a conspecific, we can assume that the animals did not have similar levels of motivation in the three groups. It is known that rats are naturally curious and highly exploratory, so the construction of a more positive environment for the Co-Test group during the experiment could have altered the motivational state of the individuals, in a way that this emotional condition would have possibly endured across the encoding phase and also emerged on the test session, 24 h later. The fact that rats performed affiliative behaviors during the test and explored the objects in physical contact with each other seems to provide evidence for this assumption.

Some additional behavioral categories were analyzed in this study. Concerning the hierarchy, there was no effect of the dominance status on the WWWhen/ELM task. Although we found no influence of this variable, it has to be stressed the importance of detailed behavioral observations of the dyads. Weiss et al. (2015, 2017), and Dorfman et al. (2016) investigated the spatial behavior of rats in the social condition and found that animals traveled together and frequently one of the rats in the dyad was leading the other. Even though the authors did not test for social dominance – and we cannot affirm that these two variables are strictly correlated –, this issue still requires further investigation in memory assessments, to rule out the possibility that some animals (such as subordinate or follower rats) were simply following the other animals or tracking their odor (such as dominant or leader rats), instead of actually exhibiting object exploration/discrimination in the task. Studies with others species have reported that the quality of relationships between individuals might lead to confounding responses, such as delayed exploration (Ryer and Olla, 1991; Brown and Laland, 2002; van Oers et al., 2005). In this respect, here we observed a complete absence of agonistic behaviors. Rats in dyads only expressed affiliative behaviors toward each other, which validates the positive effect of the social environment.

Besides the social environment itself, other factors might be related to the enhanced performance observed in the Co-Test group. Context – including an animals’s internal context – is considered an important retrieval cue (Roberts, 2019). In this sense, the constant presence of a conspecific in all experimental procedures might have enhanced the episodic ability for these rats, given that the introduction of the objects used in the task was the only change in the experimental environment. Thus, for these animals, the novelty in the experimental setting came solely from the objects themselves, what might have improved attentional processes. In the context of episode configuration, some interesting assumptions arise. Sawangjit et al. reported a study about the effects of sleep on memory formation, in which rats were exposed to a conspecific in different positions of a radial arm maze, and found that social and spatial aspects were bound into a single episode (Sawangjit et al., 2017). Accordingly, it is reasonable to assume an integrated cognitive representation of the physical and social information in our experiment, where the dyad-mate forms part of the episode together with the features of the task. Assuming the perspective of a broader episodic context, it has also been proposed an interplay between hippocampus and cortical areas in detecting physical boundaries and spatiotemporal context shifts, in order to separate elements of experience and promote a clustering of stimulus into events (Brunec et al., 2018). Consequently, keeping the dyad-mates together during all the experimental phases would avoid triggering episodic memory divergence, thus facilitating the cognitive processing in the WWWhen/ELM task the for the Co-Test group. On the other hand, in our experimental design, the Co-Hab rats underwent conjunctive habituation sessions and performed the task alone. Indeed, we could not support the hypothesis that joint habituation to the apparatus has beneficial effects on the ELM task. Rather, the data shows that the social context can provide an improvement in cognitive performance only if the animals are kept together during all the experimental procedures. Thus, we can suppose that the disruption of the social environment for the Co-Hab group was followed by alterations in emotionality and exploratory activity. Therefore, it is clear that the results observed in the test incorporate many factors, which deserve to be better investigated in future research.

Whereas the behaviors evaluated suggest a facilitating effect associated with the social context, we understand that it is not yet possible to have an accurate comprehension of the cognitive mechanisms involved in our findings. Our data implies that the presence of a cagemate seems to enhance the construction of episodic-like memory, either by promoting a better encoding or by altering the individual’s motivation to explore the objects in the ELM task, thus avoiding an expression of latent learning. Both possibilities are equally possible to play a role when animals undergo the task. Based on our current experimental design, we cannot make assumptions about the social influence on the specific processes of acquisition, consolidation, and retrieval, but this is an interesting question to address in the future. Furthermore, the fact that animals interact with each other provides opportunity for the emergence of social learning processes in the task. Previously, Lipina and Roder assumed a co-learning effect in pairs of mice involved in a spatial object recognition test (Lipina and Roder, 2013). In spite of the lack of evidence concerning the active engagement of the animals in their report, the social learning effect was evident on the task. Likewise, we believe that our experimental design is compatible with a process of stimulus enhancement, a type of social facilitation where “one animal draws another animal’s attention to the stimuli with which the former animal was interacting” (Heyes, 1994). In future studies, it would be interesting to implement changes in the research design to explore the issue of social learning in more detail, in order to identify possible behavioral cues provided by one rat to convey information to the dyad-mate or even to recognize the mechanism involved.

Overall, based on our research design, it is also relevant to evaluate an alternative explanation to the social phenomenon *per se*. It has been shown that rodents exposed to enriched environments present increased neurogenesis rates and improved biobehavioral responses – in comparison to animals raised in standard laboratory cages (Kempermann et al., 1997; Neal et al., 2018). Although the aforementioned results comprise long-term studies, it would be plausible to ask whether the factors underlying the differential performance of the Co-Test group arose from the actual presence of a conspecific or from some sort of environmental enrichment outcome, due to the dyadic coupling. We cannot entirely exclude, for instance, the possibility that a similar effect in motivation or anxiety reduction would arise applying a standard environmental enrichment protocol in the memory assessment, instead of using the social setting. Thus, it would be premature to claim that our study demonstrates a unique effect from the social environment. Our experiment does, however, suggest that the presence of a cagemate is a cognitive enhancer for this type of memory. Additionally, we argue that the social environment is a relevant domain associated to information processing about the world in social species. The existence of social bonds among rats not only has consequences on their affective/drive states, but it has also an adaptive function – a good example is the behavior of foraging and feeding in groups, which is a way of optimize resources and provide security (such as the avoidance of poisonous items) (Whishaw and Kolb, 2005).

The investigation of episodic-like memory in rodents can be done through different protocols. Experiments based on the spontaneous exploration of objects have some advantages, such as the measures used to evaluate temporal, spatial and integrative memory. How rats discriminate temporally the objects is an important issue relative to episodic-like memory recall. It is important to note that rats can detect temporal order of objects presentation with a 24 h delay (Mitchell and Laiacona, 1998; Barbosa et al., 2010, 2012). It seems unlikely that the trace for sample trial 1 (25 h) is weaker than that for sample trial 2 (24 h). Furthermore, Inostroza et al. (2013) showed that rats did not forget objects from sample trial 01, since animals explored more a novel object than a copy from either sample 01 or sample 02. Taken together, it seems plausible that animal’s performance rely on recollection rather than familiarity. However, we cannot rule out that animals used a familiarity strategy to discriminate temporally the objects. Regarding why A1 is more explored than A2, this is indeed unexpected at a first sight. We can interpret this as spatial novelty is not dissociated from temporal information, since animals show an inverse pattern to recent objects (B2 > B1). Chao et al. (2020) raised an interesting hypothesis about this phenomenon. It should be noted that two objects in the sample trial 02 occupy new places relative to the first sample, which could activate a trace for place at this moment. In the test trial, old displaced object (A2) is placed at one of these locations activated recently, and old stationary (A1) had always being located in a spot previously occupied. Therefore, A2 novelty induced by displacement could be nullified by the trace activated in the second sample trial. Although the protocol limitations, this is, to the best of our knowledge, the only task that hypothetically evaluates ELM integration.

Our approach provides a social perspective to the field of episodic-like memory. Taken together, our results suggest a refinement in the methodological approach, considering the relevance of tasks that fits the behavioral criteria for ELM investigation and offers scope for social interactions. Considering our data regarding the role of motivation and a low anxiety-like state – or a concurrent effect of both variables – it would be easier to replicate the behavioral pattern expected for the WWWhen/ELM task. Besides, studies developed in a more naturalistic setting not only help the understanding of the neural basis of episodic memory but are of great value for an evolutionary investigation. A recent work of Panoz-Brown et al. indicate that rats are able to replay a stream of different episodic memories, and the authors highlight that this capacity is quite old in the evolutionary timescale (Panoz-Brown et al., 2018). As it seems, accumulating knowledge in this area is helping to clarify the evolutionary drivers of this cognitive ability.

In conclusion, our findings indicate that rats tested in a social environment can form episodic-like memories that last one day. We propose that the presence of a conspecific decreases emotional reactivity to the experimental setting and stimulates exploration. We think that our data holds potential for the design of tasks that might be used as animal models of episodic-like memory in future research.

## Data Availability Statement

The raw data supporting the conclusions of this article will be made available by the authors, without undue reservation.

## Ethics Statement

The animal study was reviewed and approved by Comissão de ética no uso de animais (CEUA).

## Author Contributions

FB conceived the project and designed the experiment. MF, PHM, and PEM performed the experiment. FB, RC-B, MF, YS, and PEM analyzed the data. RC-B, FB, and YS wrote the manuscript. All authors contributed to the article and approved the submitted version.

## Conflict of Interest

The authors declare that the research was conducted in the absence of any commercial or financial relationships that could be construed as a potential conflict of interest.
